# A guide to selecting high-performing antibodies for Heterogeneous nuclear ribonucleoproteins A2/B1(hnRNP A2/B1) (UniProt ID: P22626) for use in western blot, immunoprecipitation, and immunofluorescence

**DOI:** 10.12688/f1000research.170886.1

**Published:** 2025-10-23

**Authors:** Vera Ruíz Moleón, Riham Ayoubi, Charles Alende, Maryam Fothouhi, Joel Ryan, Sara González Bolívar, Donovan Worrall, Thomas M Durcan, Claire M Brown, Vincent Francis, Peter S McPherson, Carl Laflamme

**Affiliations:** 1 Department of Neurology and Neurosurgery, Montreal Neurological Institute-Hospital,Structural Genomics Consortium, ABIF consortium, Montreal, Québec, Canada; 2 McGill University, Montreal, Québec, Canada

**Keywords:** P22626, HNRNPA2B1, Heterogeneous nuclear ribonucleoproteins A2/B1, antibody characterization, antibody validation, western blot, immunoprecipitation, immunofluorescence

## Abstract

Heterogeneous nuclear ribonucleoprotein A2/B1 (hnRNP A2/B1) is a key RNA-binding protein involved in alternative splicing, mRNA transport, and local translation processes essential for neuronal development and synaptic plasticity. Here we have characterized eight hnRNP A2/B1 commercial antibodies for western blot, immunoprecipitation, and immunofluorescence using a standardized experimental protocol based on comparing read-outs in knockout cell lines and isogenic parental controls. These studies are part of a larger, collaborative initiative seeking to address antibody reproducibility issues by characterizing commercially available antibodies for human proteins and publishing the results openly as a resource for the scientific community. While the use of antibodies and protocols vary between laboratories, we encourage readers to use this report as a guide to select the most appropriate antibodies for their specific needs.

## Introduction

hnRNP A2/B1 is an essential RNA-binding protein that regulates mRNA splicing, stability, and axonal transport in neurons. ^1^ Highly enriched in the central nervous system, hnRNP A2/B1 regulates the transport of mRNAs to dendrites and axons, where localized translation supports rapid, activity-dependent responses. ^2^ Pathogenic mutations and misregulation of hnRNP A2/B1 have been linked to neurodegenerative diseases, including amyotrophic lateral sclerosis (ALS), frontotemporal dementia (FTD), and multiple system atrophy. Notably, mutations in the *HNRNPA2B1* gene can lead to prion-like aggregation and cytoplasmic mislocalization, disrupting normal RNA metabolism and protein homeostasis. ^3^ hnRNP A2/B1 also interacts with stress granules and shares functional similarities with other ALS-linked proteins such as TDP-43 and FUS. ^4^ Its dual role in maintaining RNA dynamics and ^5^ vulnerability and suggests potential therapeutic avenues targeting RNA-protein homeostasis.

This research is part of a broader collaborative initiative in which academics, funders and commercial antibody manufacturers are working together to address antibody reproducibility issues by characterizing commercial antibodies for human proteins using standardized protocols, and openly sharing the data. ^5^ It consists of identifying human cell lines with adequate target protein expression and the development/contribution of equivalent knockout (KO) cell lines, followed by antibody characterization procedures using most commercially available renewable antibodies against the corresponding protein. ^5^ Here we characterized eight commercial hnRNP A2/B1 antibodies, selected and donated by participant antibody manufacturers, for use in western blot, immunoprecipitation, and immunofluorescence (also referred to as immunocytochemistry), enabling biochemical and cellular assessment of hnRNP A2/B1 properties and function. The platform for antibody characterization used to carry out this study was endorsed by a committee of industry academic representatives. The standardized consensus antibody characterization protocols are openly available on Protocol Exchange.

The authors do not engage in result analysis or offer explicit antibody recommendations. Our primary aim is to deliver top-tier data to the scientific community, grounded in Open Science principles. This empowers experts to interpret the characterization data independently, enabling them to make informed choices regarding the most suitable antibodies for their specific experimental needs. Guidelines on how to interpret antibody characterization data found in this study are featured on the YCharOS gateway ^6^ and in Table 4 of this data note. ^5^

**Table 1.** Summary of the cell lines used.

Institution	Catalog number	RRID (Cellosaurus)	Cell line	Genotype
Horizon Discovery	C631	CVCL_Y019	HAP1	WT
Horizon Discovery	HZGHC007378c010	CVCL_C9EQ	HAP1	*HNRNPA2B1* KO

**Table 2.** Summary of the hnRNP A2/B1 antibodies tested.

Company	Catalog number	Lot number	RRID (Antibody Registry)	Clonality	Clone ID	Host	Concentration (μg/μl)	Vendors recommended applications
Bio-Techne	NB120-6102 *	B1	AB_790226	monoclonal	DP3B3	mouse	1	Wb, IP, IF
Bio-Techne	NBP2-80777 *	B1	AB_2943694	monoclonal	DP3B3	mouse	1	Wb, IP, IF
Cell Signaling Technology	9304 *	2	AB_10694208	monoclonal	2A2	mouse	n/a	Wb
Genetex	GTX114475	40618	AB_11165592	polyclonal	-	rabbit	0.75	Wb, IF
Genetex	GTX127928	44573	AB_2616069	polyclonal	-	rabbit	1.09	Wb, IP, IF
Genetex	GTX637305 **	44837	AB_2943695	recombinant-mono	HL1706	rabbit	1	Wb, IF
Proteintech	67445-1-Ig *	10027542	AB_2882679	monoclonal	3H6F7	mouse	1.8	Wb, IF
Thermo Fisher scientific	PA5-81963	YE3914206A	AB_2789124	polyclonal	-	rabbit	0.1	Wb

Wb = Western blot; IF = Immunofluorescence; IP = Immunoprecipitation,

** = Recombinant antibody,

* = Monoclonal antibody, NA = Not available.

**Table 3.** Table of secondary antibodies used.

Company	Secondary antibody	Catalog number	RRID (Antibody Registry)	Clonality	Concentration (μg/μL)	Working concentration (μg/mL)
Thermo Fisher Scientific	HRP-Goat Anti-Rabbit Antibody (H+L)	65-6120	AB_2533967	polyclonal	1.0	0.2
Thermo Fisher Scientific	HRP-Goat Anti-Mouse Antibody (H+L)	62-6520	AB_2533947	polyclonal	1.5	0.75
Millipore Sigma	Protein A, HRP conjugate	P8651	NA	polyclonal	-	0.3
Thermo Fisher Scientific	Alexa Fluor™ 555-Goat anti-Rabbit IgG (H+L)	A-21429	AB_2535850	polyclonal	2.0	0.5
Thermo Fisher Scientific	Alexa Fluor™ 555-Goat anti-Mouse IgG (H+L)	A-21424	AB_141780	polyclonal	2.0	0.5

**Table 4.** Illustrations to assess antibody performance in all western blot, immunoprecipitation and immunofluorescence.

Western blot	Immunoprecipitation	Immunofluorescence
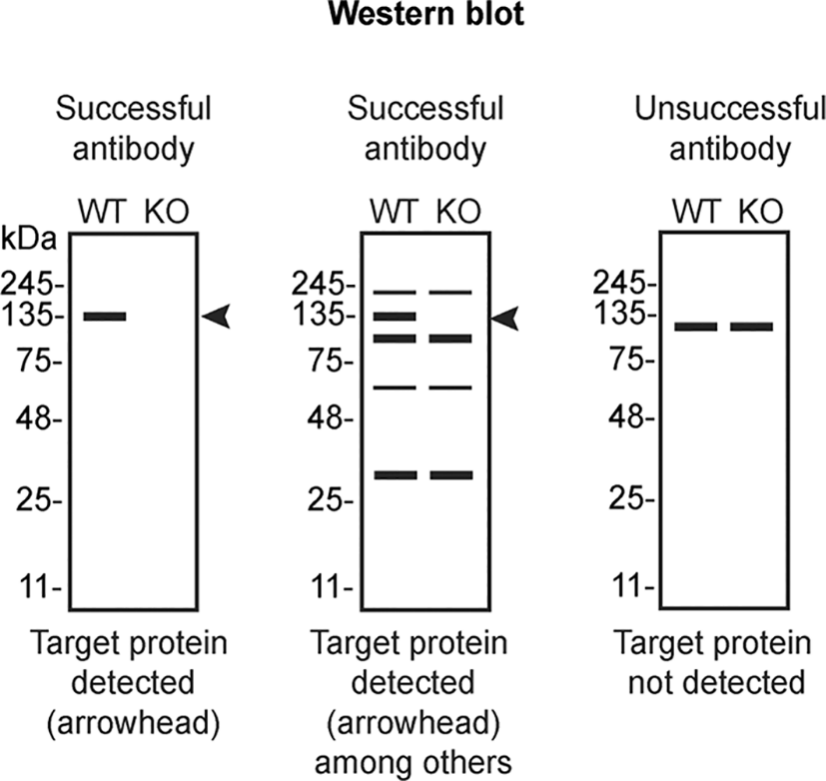	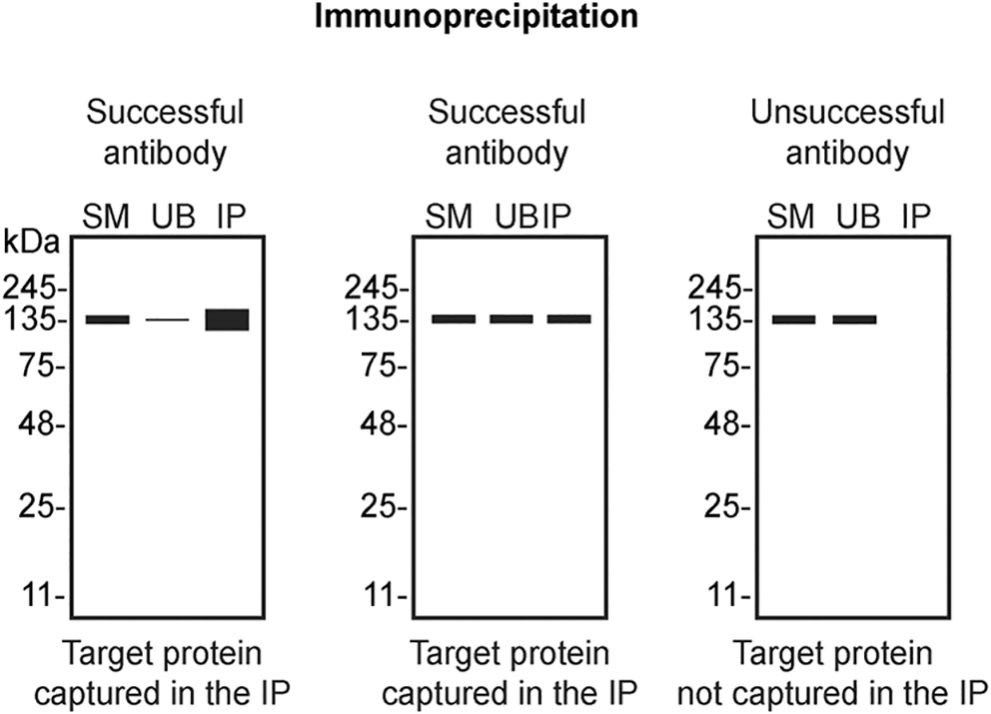	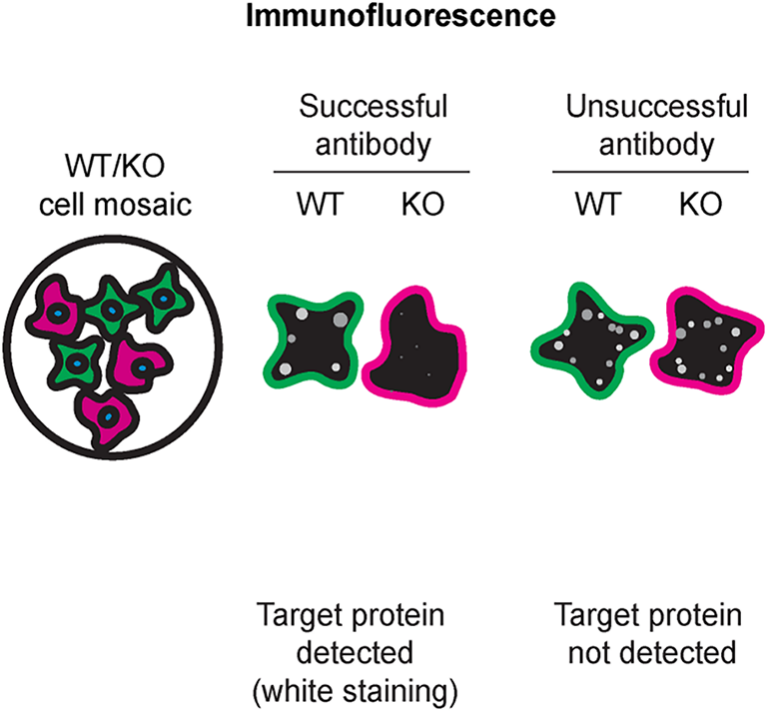

This table has been reproduced with permission from Ayoubi et al., elife, 2023. ^9^

## Results and discussion

Our standard protocol involves comparing readouts from wild type (WT) and KO cells. ^7, 8^ The first step was to identify a cell line(s) that expresses sufficient levels of a given protein to generate a measurable signal using antibodies. To this end, we examined the DepMap (Cancer Dependency Map Portal, RRID:SCR_017655) transcriptomics database to identify all cell lines that express the target at levels greater than 2.5 log _2_ (transcripts per million “TPM” + 1), which we have found to be a suitable cut-off. ^9^ The HAP1 expresses the *HNRNPA2B1* transcript at 1.01 log _2_ TPM+1. A *HNRNPA2B1* KO HAP1 cell line was obtained from Horizon Discovery ( Table 1). Moreover, as seen on DepMap, the HAP1 does not carry mutations in the *HNRNPA2B1* that could affect antibody–epitope binding.

**Figure 1.**
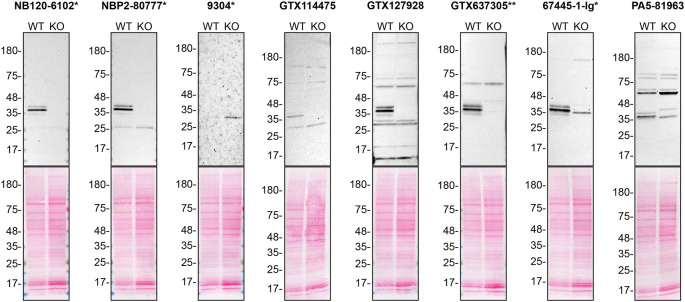
hnRNP A2/B1 antibody screening by western blot. Lysates of HAP1 WT and *HNRNPA2B1* KO were prepared, and 30 μg of protein were processed for western blot with the indicated hnRNP A2/B1 antibodies. The Ponceau stained transfers of each blot are presented to show equal loading of WT and KO lysates and protein transfer efficiency of acrylamide gels to the nitrocellulose membrane. Antibody dilutions were chosen according to the recommendations of the antibody supplier. Antibody dilution used: NB120-6102* at 1/1000; NBP2-80777* at 1/1000; 9304* at 1/1000; GTX114475 at 1/1000; GTX127928 at 1/1000; GTX637305** at 1/1000; 67445-1-Ig* at 1/1000; PA5-81963 at 1/1000. Predicted band size: 37 kDa. *=monoclonal antibody, **=recombinant antibody.

To screen all eight by western blot, WT and *HNRNPA2B1* KO protein lysates were ran on SDS-PAGE, transferred onto nitrocellulose membranes, and then probed with eight hnRNP A2/B1 antibodies in parallel ( Figure 1).

**Figure 2.**
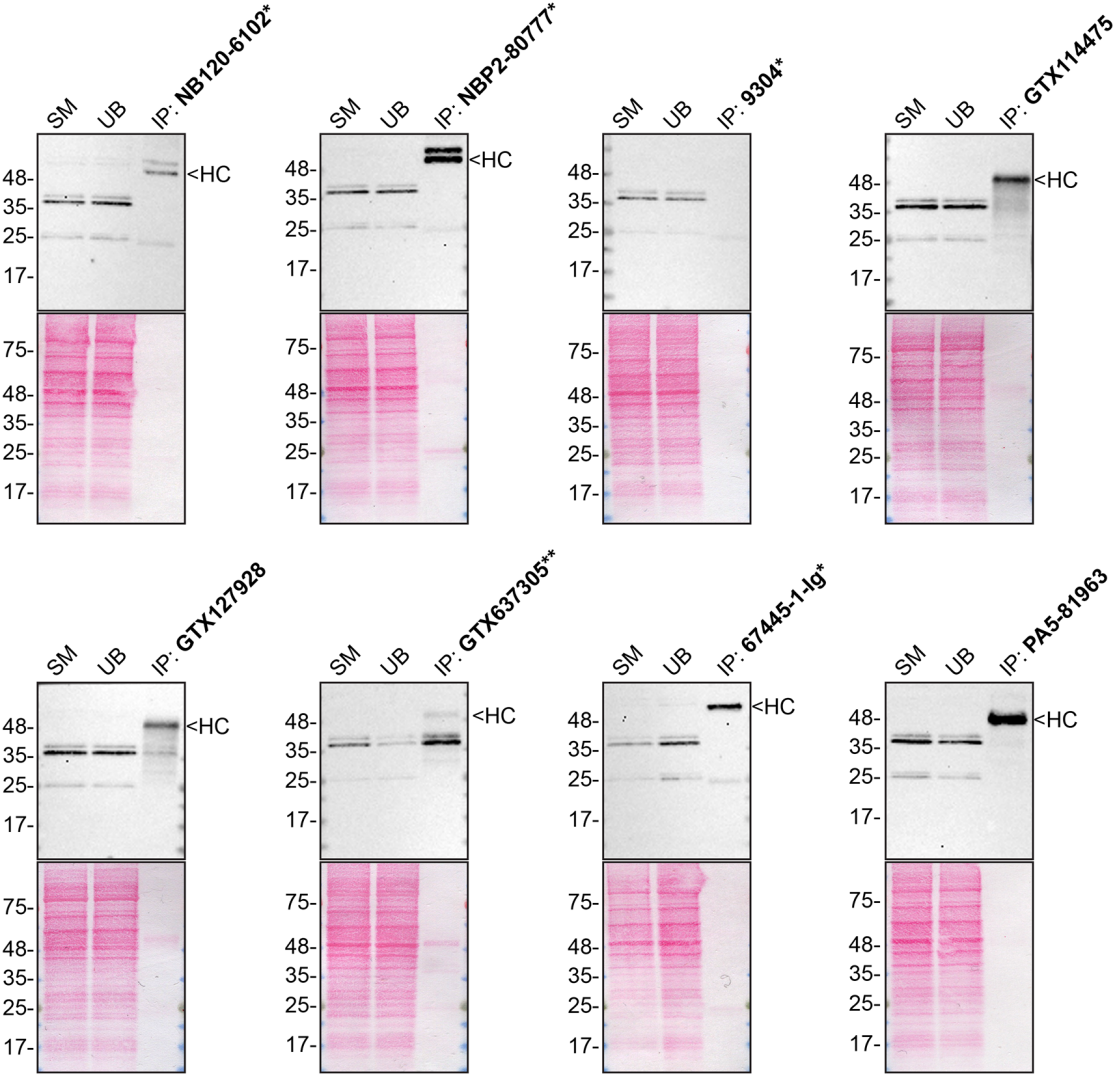
hnRNP A2/B1 antibody screening by immunoprecipitation. HAP1 WT lysates were prepared, and immunoprecipitation was performed using 2 mg of lysate and 2.0 μg of the indicated hnRNP A2/B1 antibodies pre-coupled to Dynabeads protein A or protein G. Samples were washed and processed for western blot with the hnRNP A2/B1 antibody NBP2-80777* was used at 1/1000. The Ponceau stained transfers of each blot are shown. SM = 4% starting material; UB = 4% unbound fraction; IP = immunoprecipitate, HC = antibody heavy chain. **= recombinant antibody, *= monoclonal antibody.

We then assessed the capability of all eight antibodies to capture hnRNP A2/B1 from HAP1 protein extracts using immunoprecipitation techniques, followed by western blot analysis. For the immunoblot step, a specific hnRNP A2/B1 antibody identified previously (refer to Figure 1) was selected. Equal amounts of the starting material (SM) and the unbound fractions (UB), as well as the whole immunoprecipitate (IP) eluates were separated by SDS-PAGE ( Figure 2).

**Figure 3.**
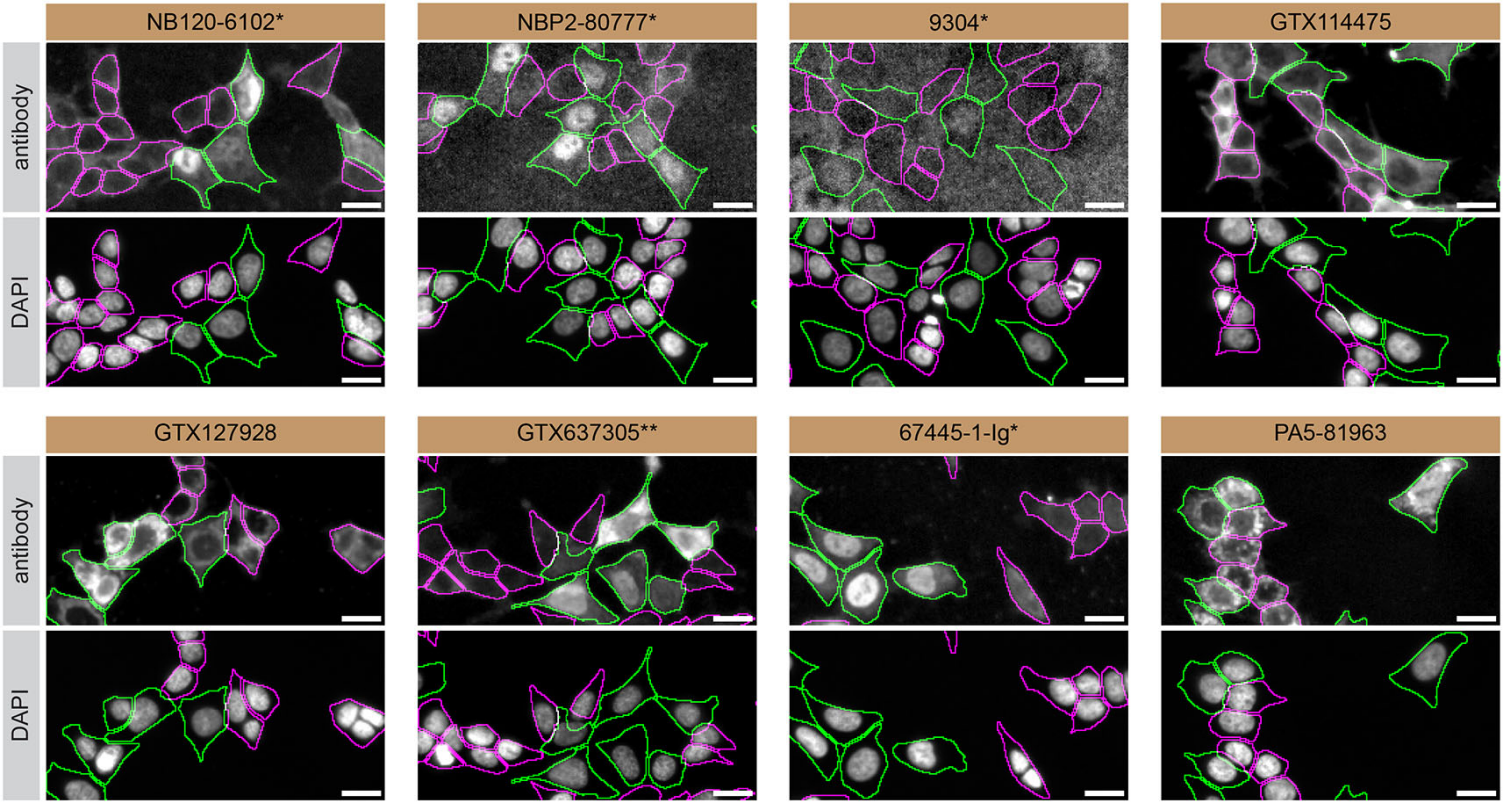
hnRNP A2/B1 antibody screening by immunofluorescence. HAP1 WT and *HNRNPA2B1* KO cells were labelled with a green or a far-red fluorescent dye, respectively. WT and KO cells were mixed and plated to a 1:1 ratio on coverslips. Cells were stained with the indicated hnRNP A2/B1 antibodies and with the corresponding Alexa-fluor 555 coupled secondary antibody including DAPI. Acquisition of the blue (nucleus-DAPI), green (WT), red (antibody staining) and far-red (KO) channels was performed. Representative images of the blue and red (grayscale) channels are shown. WT and KO cells are outlined with green and magenta dashed line, respectively. When an antibody was recommended for immunofluorescence by the supplier, we tested it at the recommended dilution. The rest of the antibodies were tested at 1 and 2 μg/ml, and the final concentration was selected based on the detection range of the microscope used and a quantitative analysis not shown here. Antibody dilution used: NB120-6102* at 1/1000; NBP2-80777* at 1/1000; 9304* at 1/1000; GTX114475 at 1/1000; GTX127928 at 1/1000; GTX637305** at 1/1000;67445-1-Ig* at 1/800; PA5-81963 at 1/100. Bars = 10 μm. *= monoclonal antibody, **= recombinant antibody.

For immunofluorescence, eight antibodies were screened using a mosaic strategy. First, HAP1 WT and *HNRNPA2B1* KO cells were labelled with different fluorescent dyes in order to distinguish the two cell lines, and the hnRNP A2/B1 antibodies were evaluated. Both WT and KO lines imaged in the same field of view to reduce staining, imaging and image analysis bias ( Figure 3). Quantification of immunofluorescence intensity in hundreds of WT and KO cells was performed for each antibody tested, and the images presented in Figure 3 are representative of this analysis. ^5^

In conclusion, we have screened eight hnRNP A2/B1 commercial antibodies by western blot, immunoprecipitation, and immunofluorescence by comparing the signal produced by the antibodies in human HAP1 WT and *HNRNPA2B1* KO cells. To assist users in interpreting antibody performanyce, Table 4 outlines various scenarios in which antibodies may perform in all three applications. ^9^ High-quality and renewable antibodies that successfully detect hnRNP A2/B1 were identified in all applications. Researchers who wish to study hnRNP A2/B1 in a different species are encouraged to select high-quality antibodies, based on the results of this study, and investigate the predicted species reactivity of the manufacturer before extending their research.

### Limitations

Inherent limitations are associated with the antibody characterization platform used in this study. Firstly, the YCharOS project focuses on renewable (recombinant and monoclonal) antibodies and does not test all commercially available hnRNP A2/B1 antibodies. YCharOS partners provide approximately 80% of all renewable antibodies, but some top-cited polyclonal antibodies may not be available through these partners. We encourage readers to consult vendor documentation to identify the specific antigen each antibody is raised against, where such information is available.

Secondly, the YCharOS effort employs a non-biased approach that is agnostic to the protein for which antibodies have been characterized. The aim is to provide objective data on antibody performance without preconceived notions about how antibodies should perform or the molecular weight that should be observed in western blot. As the authors are not experts in hnRNP A2/B1, only a brief overview of the protein's function and its relevance in disease is provided. hnRNP A2/B1 experts are invited to analyze and interpret observed banding patterns in western blots and subcellular localization in immunofluorescence.

Thirdly, YCharOS experiments are not performed in replicates primarily due to the use of multiple antibodies targeting various epitopes. Once a specific antibody is identified, it validates the protein expression of the intended target in the selected cell line, confirms the lack of protein expression in the KO cell line and supports conclusions regarding the specificity of the other antibodies. All experiments are performed using master mixes, and meticulous attention is paid to sample preparation and experimental execution. In IF, the use of two different concentrations serves to evaluate antibody specificity and can aid in assessing assay reliability. In instances where antibodies yield no signal, a repeat experiment is conducted following titration. Additionally, our independent data is performed subsequently to the antibody manufacturers internal validation process, therefore making our characterization process a repeat.

Lastly, as comprehensive and standardized procedures are respected, any conclusions remain confined to the experimental conditions and cell line used for this study. The use of a single cell type for evaluating antibody performance poses as a limitation, as factors such as target protein abundance significantly impact results. Additionally, the use of cancer cell lines containing gene mutations poses a potential challenge, as these mutations may be within the epitope coding sequence or other regions of the gene responsible for the intended target. Such alterations can impact the binding affinity of antibodies. This represents an inherent limitation of any approach that employs cancer cell lines.

## Method

The standardized protocols used to carry out this KO cell line-based antibody characterization platform was established and approved by a collaborative group of academics, industry researchers and antibody manufacturers. The detailed materials and step-by-step protocols used to characterize antibodies in western blot, immunoprecipitation and immunofluorescence are openly available on Protocols.io ( protocols.io/view/a-consensus-platform-for-antibody-characterization ). ^5^ Brief descriptions of the experimental setup used to carry out this study can be found below.

### Cell lines and antibodies

The cell lines, primary and secondary antibodies used in this study are listed in Table 1, 2, and 3, respectively. To ensure consistency with manufacturer recommendations and account for proprietary formulations (where antibody concentrations are not disclosed), antibody usage is reported as dilution ratios rather than absolute concentrations. To facilitate proper citation and unambiguous identification, all cell lines and antibodies are referenced with their corresponding Research Resource Identifiers (RRIDs). ^10, 11^ HAP1 KO clone corresponding to the *HNRNPA2B1* was purchased from Horizon Discovery commercially. All cell lines used in this study were regularly tested for mycoplasma contamination and were confirmed to be mycoplasma-free.

### Antibody screening by western blot

HAP1 WT and *HNRNPA2B1* KO cells were collected in RIPA buffer (25mM Tris-HCl pH 7.6, 150mM NaCl, 1% NP-40, 1% sodium deoxycholate, 0.1% SDS) (Thermo Fisher Scientific, cat. number 89901) supplemented with 1x protease inhibitor cocktail mix (MilliporeSigma, cat. number P8340). Lysates were sonicated briefly and incubated 30 min on ice. Lysates were spun at ~110,000 *x g* for 15 min at 4°C and equal protein aliquots of the supernatants were analyzed by SDS-PAGE and western blot. BLUelf prestained protein ladder (GeneDireX, cat. number PM008-0500) was used.

Western blots were performed with precast midi 4-20% Tris-Glycine polyacrylamide gels (Thermo Fisher Scientific, cat. number WXP42012BOX) ran with Tris/Glycine/SDS buffer (Bio-Rad, cat. number 1610772), loaded in Laemmli loading sample buffer (Thermo Fisher Scientific, cat. number AAJ61337AD) and transferred on nitrocellulose membranes. Proteins on the blots were visualized with Ponceau S staining (Thermo Fisher Scientific, cat. number BP103-10) which is scanned to show together with individual western blot. Blots were blocked with 5% milk for 1 hr, and antibodies were incubated O/N at 4°C with 5% milk in TBS with 0,1% Tween 20 (TBST) (Cell Signalling Technology, cat. number 9997). Following three washes with TBST, the peroxidase conjugated secondary antibody was incubated at a dilution of ~0.2 μg/ml in TBST with 5% milk for 1 hr at room temperature followed by three washes with TBST. Membranes were incubated with Pierce ECL (Thermo Fisher Scientific, cat. number 32106) prior to detection with the iBright™ CL1500 Imaging System (Thermo Fisher Scientific, cat. number A44240).

### Antibody screening by immunoprecipitation

Antibody-bead conjugates were prepared by adding 2 μg to 500 μl of Pierce IP Lysis Buffer from Thermo Fisher Scientific (cat. number 87788) in a microcentrifuge tube, together with 30 μl of Dynabeads protein A- (for rabbit antibodies) or protein G- (for mouse antibodies) (Thermo Fisher Scientific, cat. number 10002D and 10004D, respectively). Tubes were rocked for ~1 h at 4°C followed by two washes to remove unbound antibodies.

HAP1 WT lysates were collected in Pierce IP buffer (25 mM Tris-HCl pH 7.4, 150 mM NaCl, 1 mM EDTA, 1% NP-40 and 5% glycerol) supplemented with protease inhibitor. Lysates were rocked 30 min at 4°C and spun at 110,000 *x g* for 15 min at 4°C. 0.5 ml aliquots at 2 mg/ml of lysate were incubated with an antibody-bead conjugate for ~1 h at 4°C. The unbound fractions were collected, and beads were subsequently washed three times with 1.0 ml of IP buffer and processed for SDS-PAGE and western blot on precast midi 4-20% Tris-Glycine polyacrylamide gels. Protein A:HRP was used as a secondary detection system at a concentration of 0.3 μg/ml.

### Antibody screening by immunofluorescence

HAP1 WT and *HNRNPA2B1* KO cells were labelled with a green and a far-red fluorescence dye, respectively (Thermo Fisher Scientific, cat. number C2925 and C34565). The nuclei were labelled with DAPI (Thermo Fisher Scientific, cat. Number D3571) fluorescent stain. WT and KO cells were plated on 96-well plate with optically clear flat-bottom (Perkin Elmer, cat. number 6055300) as a mosaic and incubated for 24 hrs in a cell culture incubator at 37°C, 5% CO _2_. Cells were fixed in 4% paraformaldehyde (PFA) (VWR, cat. number 100503-917) in phosphate buffered saline (PBS) (Wisent, cat. number 311-010-CL). Cells were permeabilized in PBS with 0,1% Triton X-100 (Thermo Fisher Scientific, cat. number BP151-500) for 10 min at room temperature and blocked with PBS with 5% BSA, 5% goat serum (Gibco, cat. number 16210-064) and 0.01% Triton X-100 for 30 min at room temperature. Cells were incubated with IF buffer (PBS, 5% BSA, 0,01% Triton X-100) containing the primary pr hnRNP A2/B1 antibodies overnight at 4°C. Cells were then washed 3 × 10 min with IF buffer and incubated with corresponding Alexa Fluor 555-conjugated secondary antibodies in IF buffer at a dilution of 1.0 μg/ml for 1 hr at room temperature with DAPI. Cells were washed 3 × 10 min with IF buffer and once with PBS.

Images were acquired on an ImageXpress micro confocal high-content microscopy system (Molecular Devices), using a 20x NA 0.95 water immersion objective and scientific CMOS cameras, equipped with 395, 475, 555 and 635 nm solid state LED lights (lumencor Aura III light engine) and bandpass filters to excite DAPI, Cellmask Green, Alexa-555 and Cellmask Red, respectively. Images had pixel sizes of 0.68 x 0.68 microns, and a z-interval of 4 microns. For analysis and visualization, shading correction (shade only) was carried out for all images. Then, maximum intensity projections were generated using 3 z-slices. Segmentation was carried out separately on maximum intensity projections of Cellmask channels using CellPose 1.0, and masks were used to generate outlines and for intensity quantification. ^12^ Figures were assembled with Adobe Illustrator.

## Author contributions

Vera Ruíz Moleón-Investigation

Riham Ayoubi-Investigation, Supervision, Writing – Original Draft Preparation

Maryam Fotouhi-Investigation

Joel Ryan-Investigation

Donovan Worrall-Investigation

Thomas M. Durcan-Investigation

Claire M. Brown-Investigation

Vincent Francis-Writing – Review & Editing

Peter S. McPherson Roles- Supervised and Funding Acquisition

Carl Laflamme- Roles: Conceptualization, Funding Acquisition, Writing – Review & Editing

All authors reviewed and approved the final version of the manuscript.

## Data Availability

Zenodo: Dataset for the hnRNP A2/B1 antibody screening study, https://doi.org/10.5281/zenodo.17144167. ^13^ Data are available under the terms of the Creative Commons Attribution 4.0 International license (CC-BY 4.0).
