# Supplementary material for: A guide to selecting high-performing antibodies for Heterogeneous nuclear ribonucleoproteins A2/B1(hnRNP A2/B1) (UniProt ID: P22626) for use in western blot, immunoprecipitation, and immunofluorescence
Source: F1000Res. 2025 Oct 23;14:1158. [Version 1] doi: 10.12688/f1000research.170886.1 (PMC12669976; doi:10.12688/f1000research.170886.1)
Supplement: Peer review history

# Reviewer report: Reviewer response for version 1

**DOI:** 10.5256/f1000research.188398.r427059

**Published:** 2025-12-01

**Authors:** Sian E. Evans

**Affiliations:** 1 University of Leicester, Leicester, England, UK

The article explores the characterisation of eight commercially available antibodies that target Heterogeneous Nuclear Ribonucleoprotein A2/B1 (hnRNP A2/B1). These antibodies have been assessed for their effectiveness in western blotting, immunoprecipitation, and immunofluorescence techniques using HAP1 wild-type (WT) and knockout (KO) cell lines. The authors include a summary table that provides details about each antibody, including the recommended applications from the vendors for the three techniques mentioned. The methods employed in the study are clearly described and properly cited. Additionally, the inclusion of Table 4, which outlines how to evaluate antibody performance, enhances the data note significantly.

Are sufficient details of methods and materials provided to allow replication by others?

Yes

Is the rationale for creating the dataset(s) clearly described?

Yes

Are the datasets clearly presented in a useable and accessible format?

Yes

Are the protocols appropriate and is the work technically sound?

Yes

Reviewer Expertise:

Translational research scientist with expertise in developing explant technologies, multi-immunofluorescence, imaging, and histology techniques, as well as molecular biology methods like western blotting and immunoprecipitation.

I confirm that I have read this submission and believe that I have an appropriate level of expertise to confirm that it is of an acceptable scientific standard.

# Reviewer report: Reviewer response for version 1

**DOI:** 10.5256/f1000research.188398.r427060

**Published:** 2025-11-05

**Authors:** Deborah Moshinsky

**Affiliations:** 1 Institute for Protein Innovation, Boston, Massachusetts, USA

The authors tested eight Heterogeneous Nuclear Ribonucleoprotein A2/B1 (hnRNP A2/B1) commercial antibodies for activity by Western Blot, immunoprecipitation, and immunofluorescence.  They noted which applications were recommended by the vendor and showed data for the results obtained.  This is part of a larger ongoing effort to obtain antibody characterization data on commercially available antibodies to assist researchers in choosing antibodies most likely to work in their system.  The standardized protocols are clearly described and referenced.  This publication should be useful to researchers in need of good hnRNP A2/B1 antibodies.

Are sufficient details of methods and materials provided to allow replication by others?

Yes

Is the rationale for creating the dataset(s) clearly described?

Yes

Are the datasets clearly presented in a useable and accessible format?

Yes

Are the protocols appropriate and is the work technically sound?

Yes

Reviewer Expertise:

Director of antibody characterization and validation

I confirm that I have read this submission and believe that I have an appropriate level of expertise to confirm that it is of an acceptable scientific standard.
